# Tumor-infiltrating CD4^+^ T cells in patients with gastric cancer

**DOI:** 10.1186/s12935-017-0489-4

**Published:** 2017-12-02

**Authors:** Long Yuan, Benling Xu, Peng Yuan, Jinxue Zhou, Peng Qin, Lu Han, Guangyu Chen, Zhenlei Wang, Zengci Run, Peng Zhao, Quanli Gao

**Affiliations:** 1 Department of General Surgery, The Affiliated Cancer Hospital of Zhengzhou University, Zhengzhou, Henan People’s Republic of China; 2 Cancer Biotherapy Center, The Affiliated Cancer Hospital of Zhengzhou University, No. 127, Dongming Road, Zhengzhou, 450008 Henan People’s Republic of China; 3 Department of Hepatobiliary and Pancreatic Surgery, The Affiliated Cancer Hospital of Zhengzhou University, Zhengzhou, Henan People’s Republic of China

**Keywords:** T lymphocytes, CD4^+^ T-cells, Gastric cancer, IFN-γ staining assay, Dysfunction

## Abstract

**Background:**

T lymphocytes play an indispensably important role in clearing virus and tumor antigen. There is little knowledge about impacts of inhibitory molecules with cytokine on tumor-infiltrating CD4^+^ T-cells in the presence of gastric cancer (GC). This study investigated the distribution of tumor-infiltrating T-cells subset and the differentiation as well as inhibitory phenotype of T-cells from blood and tissues of GC patients.

**Materials and methods:**

Patients with GC diagnosed on the basis of pre-operative staging and laparotomy findings were approached for enrollment between 2014 and 2015 at the Affiliated Cancer Hospital of Zhengzhou University, China. Phenotypic analysis based on isolation of tumor-infiltrating lymphocytes and intracellular IFN-γ staining assay is conducted. Statistical analysis is performed to show significance.

**Results:**

The results showed that the percentage of CD4^+^ T-cells among CD3^+^ cells in tumors was significantly higher than that in the matched paraneoplastic tissue. CD4^+^ CD25^high^ CD127^low^ regulatory T-cells (Tregs), PD-1^+^, Tim-3^+^, and PD-1^+^ Tim-3^+^ cells were up-regulated on tumor infiltrating T-cells from patients with GC compared to their expressions on corresponding peripheral blood and peritumoral T-cells. Blockades of PD-1^+^ and Tim-3^+^ were effective in restoring tumor infiltrating T-cells’ production of interferon-gamma (IFN-γ). Combined PD-1^+^ and Tim-3^+^ inhibition had a synergistic effect on IFN-γ secretion by CD4^+^ T-cells.

**Conclusion:**

The results suggested that the composition, inhibitors, and location of the immune infiltrate should be considered when evaluating antitumor immunotherapy. A new insight into the mechanisms underlying T cell dysfunction is provided.

**Electronic supplementary material:**

The online version of this article (10.1186/s12935-017-0489-4) contains supplementary material, which is available to authorized users.

## Background

Gastric cancer (GC), one of the leading causes for worldwide mortality, is considered as the second most common cancer in China [1, 2]. Although the clinical management for GC is diverse, the prognosis of GC remains poor [3]. The mechanism of clinical management is supposed to include increasing regulatory T-cells (Tregs), losing tumor antigen expression, and enhancing tumor expression of inhibitory ligand [4–7]. Reports proved that the depletion of Tregs could not enhance the efficacy of primary therapies in some cancers, which suggests that T cells may work in synergy with other mechanisms to suppress anti-tumor immunity [8–10].

Investigations showed that T-cell dysfunction in chronic virus infection and human tumor growth was related to the up-regulation of inhibitory molecules such as programmed death 1 (PD-1), T-cell immunoglobulin, and mucin-domain-containing molecule 3 (Tim-3) [11–14]. It provides a significant scheme for cancer treatment that invalidates these inhibitory pathways to resume exhausted T-cells. However, only 19.5% of GC patients respond to PD-1 inhibition [15].

Currently, there is little detail of T-cell subsets resident within gastric cancer tissues or the expression patterns in this microenvironment corresponding to those observed in paraneoplastic tissue or in the peripheral blood of GC patients. Moreover, the role of PD-1^+^, Tim-3^+^, and Tregs in the development and maintenance of tumor-infiltrating T-cell dysfunction in GC patients need to be investigated. Thus, it is crucial to discover the complex mechanisms for inducing T-cell dysfunction in GC patients by exploring the subset composition and functional properties of tumor-infiltrating T-cells.

In this study, we investigated the distribution of T-cells subset, the differentiation and inhibitory phenotype of T-cells from blood and tissues of GC patients. The results demonstrated that the T-cell subsets resident within GC is different from that in paraneoplastic tissue and blood, and the increase in Tim-3^+^ PD-1^+^ CD4^+^ T-cells in tumor tissues was found to correlate with the clinical cancer stage and Tregs. These data as well as the corresponding results and the methods provide a new immunotherapeutic approach for the clinical management of GC.

## Materials and methods

### Patients

Patients with GC diagnosed on the basis of pre-operative staging and laparotomy findings were approached for enrollment between 2014 and 2015 at the Department of Surgery, the Affiliated Cancer Hospital of Zhengzhou University, China. Thirty-one patients diagnosed with primary GC without previously treatment were qualified in this study. Peripheral blood samples were collected from each patient before treatment. The clinical stage was classified according to the American Joint Committee on Cancer (AJCC) Staging Manual, Seventh Edition (2010). The clinicopathological characteristics of age, gender, and histological grade at the time of blood sample collection were recorded in Table 1.

**Table 1:** Characteristics of patients

Variable	Category	Numbers
Total patients (n)		31
Median age in years (range)		60 (37–79)
Gender	Male	21
	Female	10
AJCC stage (2010)	I/II	12
	III	19
Histologic differentiation	Well/moderate	13
	Poor	18
Weight loss (%)	None	4
	≤ 10	16
	> 10	11
Smoking status	Current	17
	Ex-smoker	4
	Never	10

### Phenotypic analysis

Fresh venous blood was collected from patients or healthy donors (HDs) with EDTA-coated vacutainer tubes. Specific anti-CD3-PE-Cy7 (BioLegend, San Diego, CA, USA) or anti-CD3-FITC (BioLegend, San Diego, CA, USA), anti-CD25-PE (BD Pharmingen™, San Diego, California, USA), anti-CD127-APC (BioLegend, San Diego, CA, USA), anti-CD4-FITC (BioLegend, San Diego, CA, USA), anti-CD8-APC (BioLegend, San Diego, CA, USA), anti-CD45RA-FITC (BioLegend, San Diego, CA, USA), anti-CCR7-PE-Cy7 (BioLegend, San Diego, CA, USA), anti-CD4-PE (BD Biosciences, Oxnard, CA), anti-PD-1-FITC (BioLegend, San Diego, CA, USA), anti-CD4-PerCP-cy5.5 (BD Biosciences, Oxnard, CA), anti-Tim-3-PE (BD Pharmingen™, San Diego, California, USA), and anti-IFN-γ-APC (eBioscience, San Diego, CA, USA) were purchased for use. The viability of the cells was assessed by a violet amine reactive dye (Invitrogen, Carlsbad, CA).

Briefly, 50 μL of blood mixed with 5 μL of each antibody was incubated on ice for 20 min in the dark. Then, 2 mL of 1× lysis buffer (BD Biosciences, Oxnard, CA) was added to each sample and incubated at room temperature for 15 min. Samples were washed with FACS buffer (5% BSA in PBS, 0.09% sodium azide), and the pellets were resuspended in 300 μL of FACS buffer. Flow cytometry was performed on a BD FACS Aria II flow cytometer, and was analyzed with FlowJo software (TreeStar Inc., Ashland, OR, USA). The values were background-corrected by control sample or fluorescence minus one (FMO). Cells without surface makers were used as control samples.

### Isolation of TILs

The fresh human tumor samples and matched paraneoplastic tissues from patients with GC were cut into pieces (3 ~ 5 mm^3^) and were treated with 1 μg/mL of collagenase (Sigma-Aldrich, St. Louis, MO, USA), 25 μg/mL of DNase (Sigma-Aldrich, St. Louis, MO, USA), and 2% fetal bovine serum in PBS at 37 °C for 1 to 1.5 h. The tissue homogenates were filtered by a 70-μm cell strainer (Falcon; BD Biosciences, Oxnard, CA) before density centrifugation. Density centrifugation was performed using Percoll density gradient, and cells at the interface between 40 and 80% discontinuous Percoll gradient were collected. The leukocyte’s viability was evaluated by trypan blue exclusion.

### Intracellular IFN-γ staining assay

To evaluate the effect of the blockade of PD-1/PD-L1/2 and Tim-3/Tim-3-L pathways on the induction of IFN-γ, TILs and T-cells in noncancerous tissues were pre-incubation with blocking antibodies for 1 h, and stimulated with 5 μg/mL of anti-CD3 (eBioscience, San Diego, CA, USA) for 6 h [14]. After incubation for another 2 h, 10 μg/mL of Brefeldin A (Sigma- Aldrich, St. Louis, MO, USA) was added to the culture medium. Cells were then stained with antibodies anti-CD3-PE-Cy7 and anti-CD4-PerCp-Cy5.5/anti-CD8-PerCp-Cy5.5, and marked with mAb against IFN-γ-APC (eBioscience, San Diego, CA, USA). Five hundred thousand events were recorded during flow cytometric analysis.

### Statistical analysis

Statistical analysis was performed by GraphPad Prism 5.0 (GraphPad Software, US). Mann–Whitney test was used to assess the differences between the study groups. A pair wised *t* test was applied to compare the expression of the inhibitory molecules in cancer, noncancerous tissues, and blood. *p* < 0.05 was considered to be statistically significant.

## Results

### T-cell subsets in GC

**Fig. 1:**
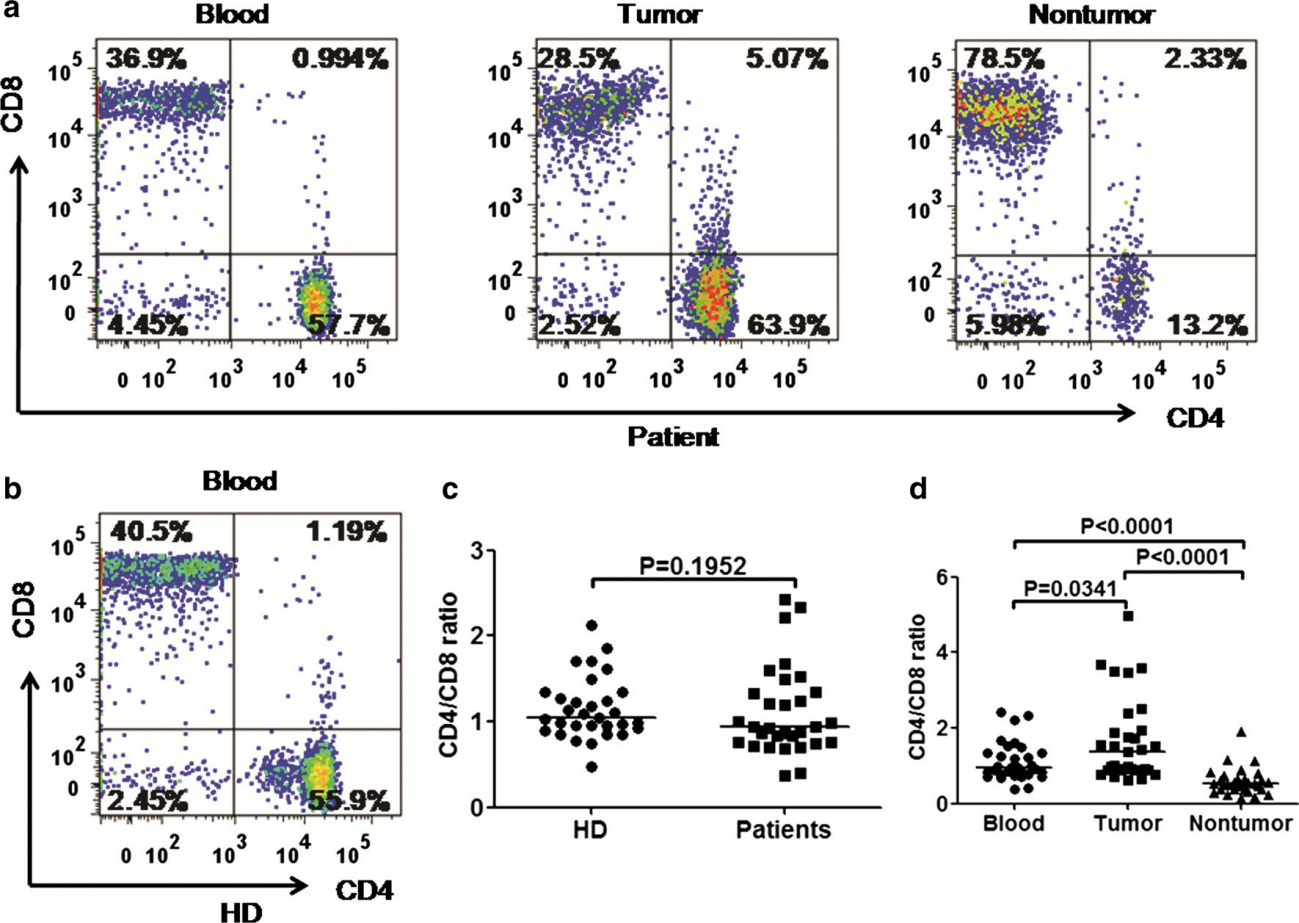
The ratio of CD4^+^/CD8^+^ T-cells among CD3^+^ T-cells in GC patientsvaried for different tissues. Representative flow cytometric dotplots of a patient and a healthy donor were shown in **a**, **b**, respectively. Pooled data showing the CD4^+^ */*CD8^+^ ratio from GC patients and HDs were illustrated in **c**. Horizontal bars depict the median of the CD4^+^/CD8^+^ ratio. The ratios of CD4^+^/CD8^+^ T-cells among CD3^+^ T-cells in GC patients between tumor-infiltrating lymphocytes and matched circulating lymphocytes or paraneoplastic tissue were shown in **d**. The *p* values were calculated using the pair wised *t* test. *p* < 0.05 was considered as statistically significant

To better understand the properties of T-cells immunity in gastric tumor masses, frequency of CD4^+^ and CD8^+^ T-cells in circulating CD3^+^ T-cells from GC patients and HDs was measured. However, no significant difference in frequency of CD4^+^ and CD8^+^ T-cells were found between GC patients and HDs (data not shown). We then further investigated the differences in frequency of CD4^+^ and CD8^+^ T-cells in CD3^+^ T-cells between TILs and matched circulating lymphocytes or paraneoplastic tissue in GC patients. Flow cytometry results showed that the tumors had a higher frequency of CD4^+^ CD8^−^ T-cells (51.3%) and a lower frequency of CD8^+^ CD4^−^ T-cells (38.4%) compared with paraneoplastic tissues or blood, which indicated a rising CD4^+^/CD8^+^ ratio in tumors (*p* = 0.0341 for tumor vs. blood, *p* < 0.0001 for tumor vs. non-tumor; Fig. 1). It was observed that the CD4^+^/CD8^+^ ratio in peripheral blood was significantly higher than that in matched paraneoplastic tissue (0.94 vs. 0.53, *p* < 0.0001, Fig. 1d). Representative flow cytometric data of a patient with GC is shown in Fig. 1a.

### Tumor-infiltrating T-cell differentiation

The balance of T-cell differentiation plays an important role in successful immune controls. Therefore, T-cell differentiation in GC patients was analyzed according to the expressions of CCR7 and CD45RA. Both the tumor-infiltrating CD4^+^ and CD8^+^ T-cells were dominated by the effector memory phenotype (TEM, CCR7^−^CD45RA^−^, 55.52% for CD4^+^, 43.11% for CD8^+^) in GC. On CD4^+^ T-cells, there are 32.67% of central memory T-cells (TCM, CCR7^+^CD45RA^−^), 6.34% of terminally differentiated T-cells (Teff, CCR7^−^CD45RA^+^) and 5.72% of naive (Tnaive, CCR7^+^CD45RA^+^, 5.72%) T-cells (Additional file 1: Figure S1), while on CD8^+^ T-cells, there are 28.5% of Teff (28.5%), 14.57% of TCM (14.57%) and 13.81% of Tnaive (13.81%) T-cells (Additional file 1: Figure S1). TEM among CD4^+^ and CD8^+^ T-cells in tumor tissues was more frequent than TCM and Tnaïve T-cells in tumor tissues. On CD4^+^ T-cells, TEM was more frequent than Teff in tumor tissues, but on CD8^+^ T-cell, there was no difference between TEM and Teff. T-cells in paraneoplastic tissue displayed similar compositions compared to TILs. Overall, patterns of CD4^+^ and CD8^+^ T-cells differentiation revealed no differences between tumor and paraneoplastic tissue. It is very interesting that the frequencies of TEM and TCM on CD4^+^ and CD8^+^ T-cells in blood were significantly lower than that in tumors, while the frequencies of Teff and Tnaïve among CD4^+^ and CD8^+^ T-cells in blood were significantly higher than those in tumors and paraneoplastic tissues (Additional file 1: Figure S1). In addition, the frequencies of TEM and TCM on CD4^+^ T-cells had no significant difference between blood and paraneoplastic tissue, but the frequencies of TEM on CD8^+^ T-cells in paraneoplastic tissues were significantly higher than those in blood (37.16% vs. 9.89%, *p* = 0.002). T cell differentiation in HDs is similar to that in HDs (data not shown).

### Tumor-infiltrating CD4^+^ CD25^high^ CD127^low^ T regulatory cells increase in GC patients

**Fig. 2:**
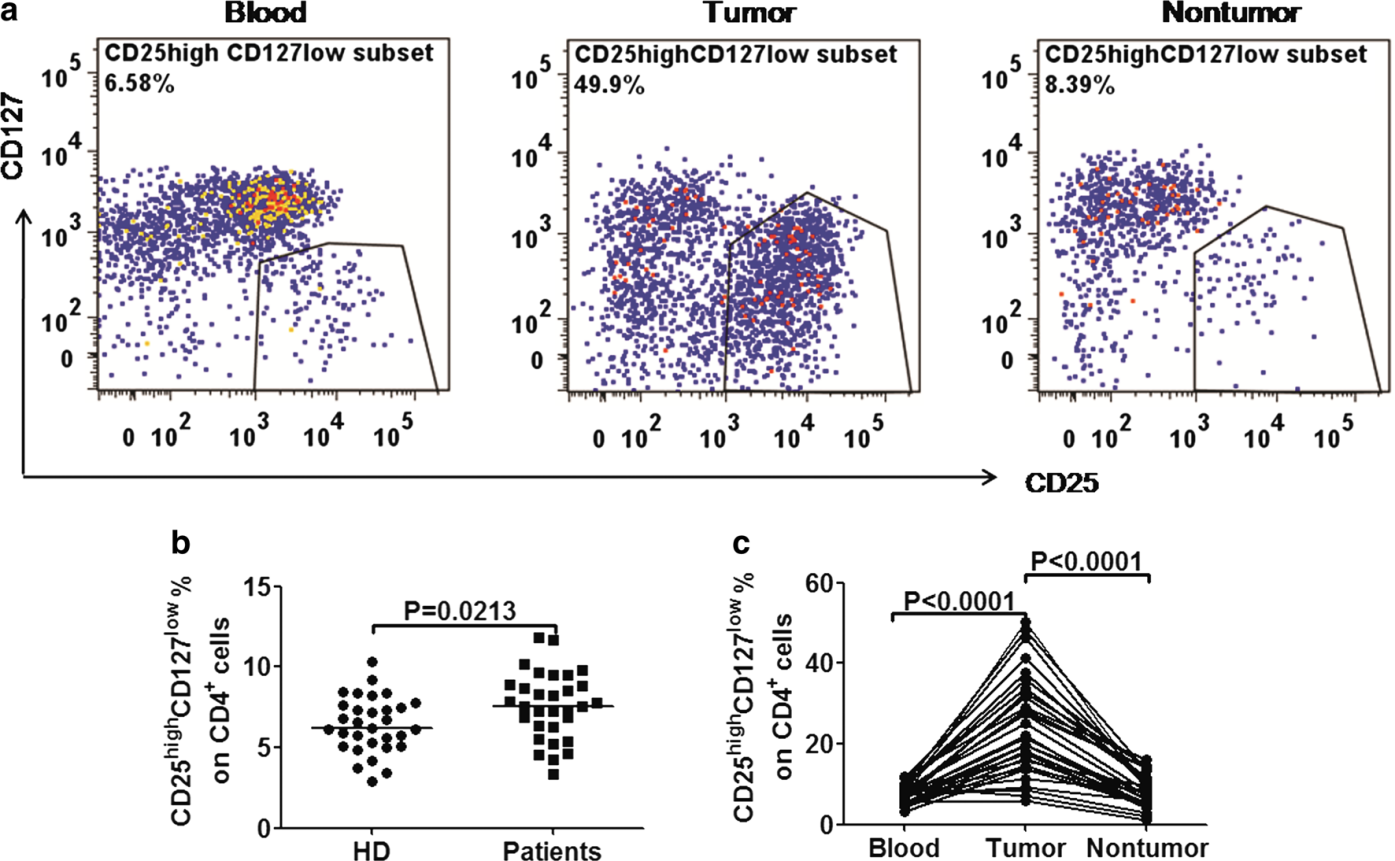
Frequencies of CD25^+^ CD127^low^ T regulatory cells among CD4^+^ T-cells in patients with GC. **a** Scatter plots of CD25^+^ CD127^low^ T regulatory cells among CD4^+^ T-cells in blood, counterparts in tumor tissue, and paraneoplastic tissue of GC patients. **b** Pooled data showing the percentage (%) of CD25^+^ CD127^low^ T regulatory cells among CD4^+^ T-cells from GC patients and HD. Horizontal bars depict the median percentage of CD25^+^ CD127^low^ T regulatory cells among CD4^+^ T-cells. **c** Pooled data showing the percentage (%) of CD25^+^ CD127^low^ T regulatory cells among CD4^+^ T-cells in blood, counterparts in paraneoplastic tissue, and tumor tissue of GC patients. A pair wised *t* test was used to compare the differences. *p* < 0.05 was considered to be statistically significant

Tumors escaped from the immune system attack via different regulatory molecules and regulatory cells, of which CD4^+^ Treg was the most extensively studied negative regulator [16]. So we analyzed the expression of CD25^high^ CD127^low^ Tregs among CD4^+^ lymphocytes in GC patients and HDs. The results were showed in Fig. 2. CD25^high^ CD127^low^ Tregs among CD4^+^ cells in GC patients were significantly higher than that in HDs (7.54% vs. 6.23%, *p* = 0.0213). CD25^high^ CD127^low^ Tregs on tumor-infiltrating CD4^+^ cells were significantly higher than those in both blood (21.54% vs. 7.54%, *p* < 0.0001) and paraneoplastic tissue (21.54% vs. 7.86%, *p* < 0.0001). The CD25^high^ CD127^low^ cells among CD4^+^ cells from noncancerous tissues was a little higher than that in blood, but showed no significant difference (7.86% vs. 7.54%, *p* = 0.1573). Representative flow cytometric data of a patient with GC is shown in Fig. 2a.

### Elevated Tim-3 and PD-1 expression on tumor-infiltrating T lymphocytes in GC patients

**Fig. 3:**
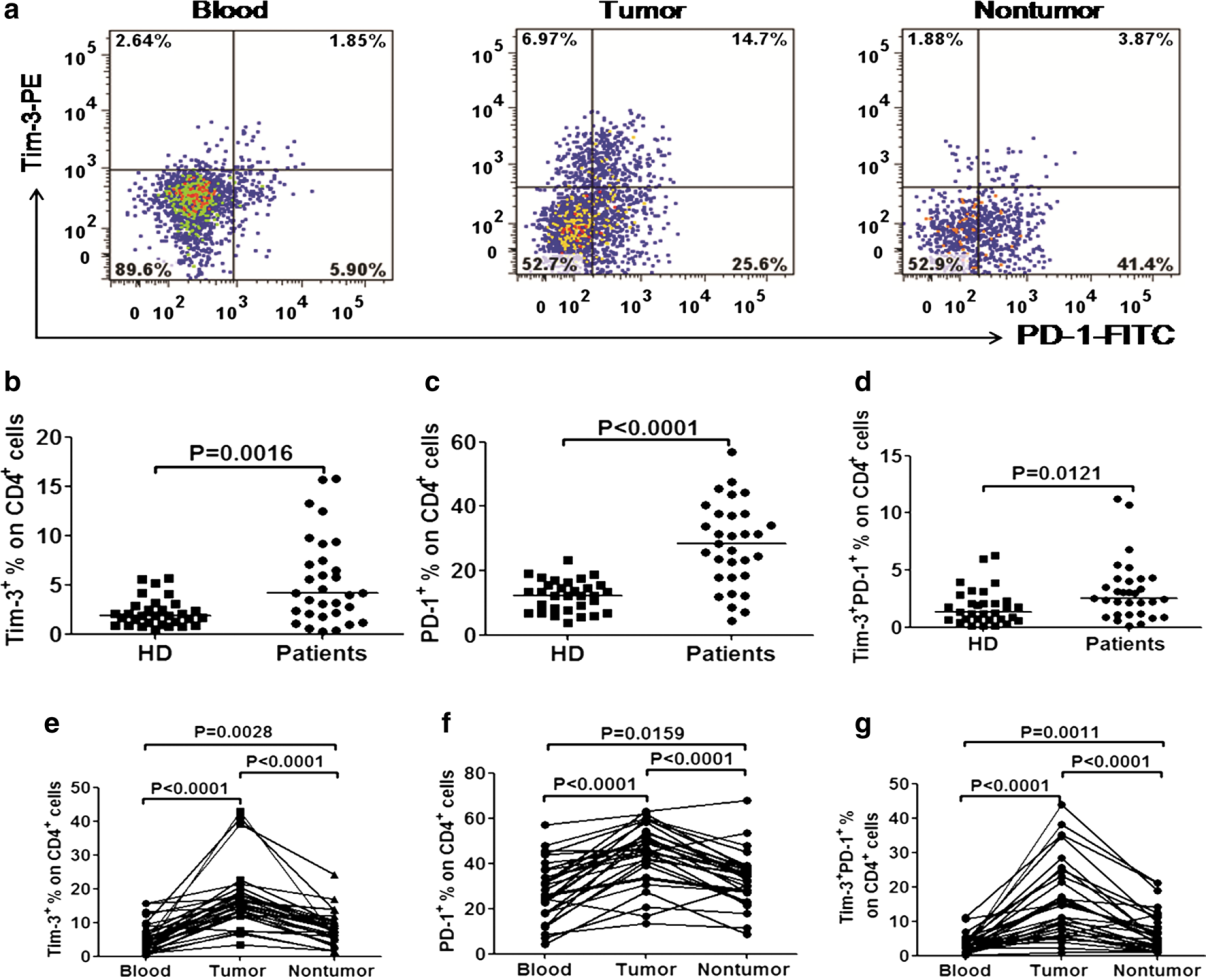
The expression of Tim-3^+^ and PD-1^+^ on CD4^+^ T-cells in GC patients. **a** Representative scatter plots illustrating the expression of Tim-3^+^ and PD-1^+^ on CD4^+^ T-cells in blood, tumor, and non-tumor. Pooled data showing the percentage (%) of Tim-3^+^ (**b**)/PD-1^+^ (**c**)/Tim-3^+^ PD-1^+^ (**d**) T-cells among CD4^+^ T-cells in blood from GC patients and HDs. Pooled data showing the percentage (%) of Tim-3^+^ (**e**)/PD-1^+^ (**f**)/Tim-3^+^ PD-1^+^ (**g**) T-cells among CD4^+^ T-cells in blood, counterparts in paraneoplastic tissue, and tumor tissue of GC patients. *p* < 0.05 was considered statistically significant

The expression of inhibitory molecules Tim-3^+^ and PD-1^+^ on T-cells in blood, paraneoplastic tissue, and tumors was analyzed. Percentages of Tim-3^+^ and PD-1^+^ were calculated as a percentage of CD4^+^/CD8^+^ positive T-cells. The representative flow cytometric dot plots were shown in Fig. 3a (of CD4^+^) and Additional file 2: Figure S2A (of CD8^+^). The frequency of Tim-3^+^ cells among CD4^+^ and CD8^+^ cells in circulation were significantly higher in GC patients than those in HDs (median, 3.5% vs. 1.7%, *p* = 0.0016, Fig. 3b for CD4^+^; 4.56% vs. 2.6%, *p* = 0.0281, Additional file 2: Figure S2B for CD8^+^). Consistent with previous studies, a significantly higher level of PD-1^+^ cells on T-cells in circulation in GC patients were observed compared with HDs (median, 25.57% vs. 12.3%, *p* < 0.0001, Fig. 3c for CD4^+^; 27.1% vs. 17.8%, *p* = 0.0014, Additional file 2: Figure S2C for CD8^+^). In our study, the median percentage of PD-1^+^ Tim-3^+^ cells among CD4^+^ and CD8^+^ T-cells in GC patients were also significantly higher than that in HDs (2.34% vs. 1.2%, *p* = 0.0121, Fig. 3d for CD4^+^; 1.45% vs. 0.98%, *p* = 0.0441, Additional file 2: Figure S2D for CD8^+^).

The difference between the expression of Tim-3 and PD-1 in blood and tissues were also explored. Compared with the matched blood, the percentage of Tim-3^+^ cells in TILs were significantly increased (16.88% vs. 5.52%, *p* < 0.0001, Fig. 3e for CD4^+^; 21.96% vs. 4.56%, *p* < 0.0001, Additional file 2: Figure S2E for CD8^+^). PD-1^+^ was also expressed at a higher level on TILs (44.7% vs. 28.05%, *p* < 0.0001, Fig. 3f for CD4^+^; 56% vs. 27.1%, *p* < 0.0001, Additional file 2: Figure S2F for CD8^+^). Similarly, both the percentages of Tim-3^+^ and PD-1^+^ cells in paraneoplastic tissue were significantly lower than those in TILs (Tim-3^+^: 8.83% vs. 16.88%, *p* < 0.0001 for CD4^+^, 6.22% vs. 21.96%, *p* < 0.0001 for CD8^+^; PD-1^+^: 33.24% vs. 44.7%, *p* < 0.0001 for CD4^+^, 45.96% vs. 56%, *p* < 0.0001 for CD8^+^). Furthermore, PD-1^+^ Tim-3^+^ cells among T-cells were significantly higher in tumor tissues than those in blood (15.47% vs. 3.17%, *p* < 0.0001, Fig. 3g for CD4^+^; 18.2% vs. 1.45%, *p* < 0.0001, Additional file 2: Figure S2G for CD8^+^) and paraneoplastic tissues (15.47% vs. 6.64%, *p* < 0.0001, Fig. 3g for CD4^+^; 18.2% vs. 3.87%, *p* < 0.0001, Additional file 2: Figure S2G for CD8^+^). Percentages of PD-1^+^ and PD-1^+^ Tim-3^+^ in T-cells of paraneoplastic tissues were all significantly higher than those in blood for both CD4^+^ and CD8^+^ T-cells.

### Ex vivo blockade of PD-1/PD-L1/2 and Tim-3/Tim-3-L pathways enhances the tumor-infiltrating T-cell IFN-γ production

**Fig. 4:**
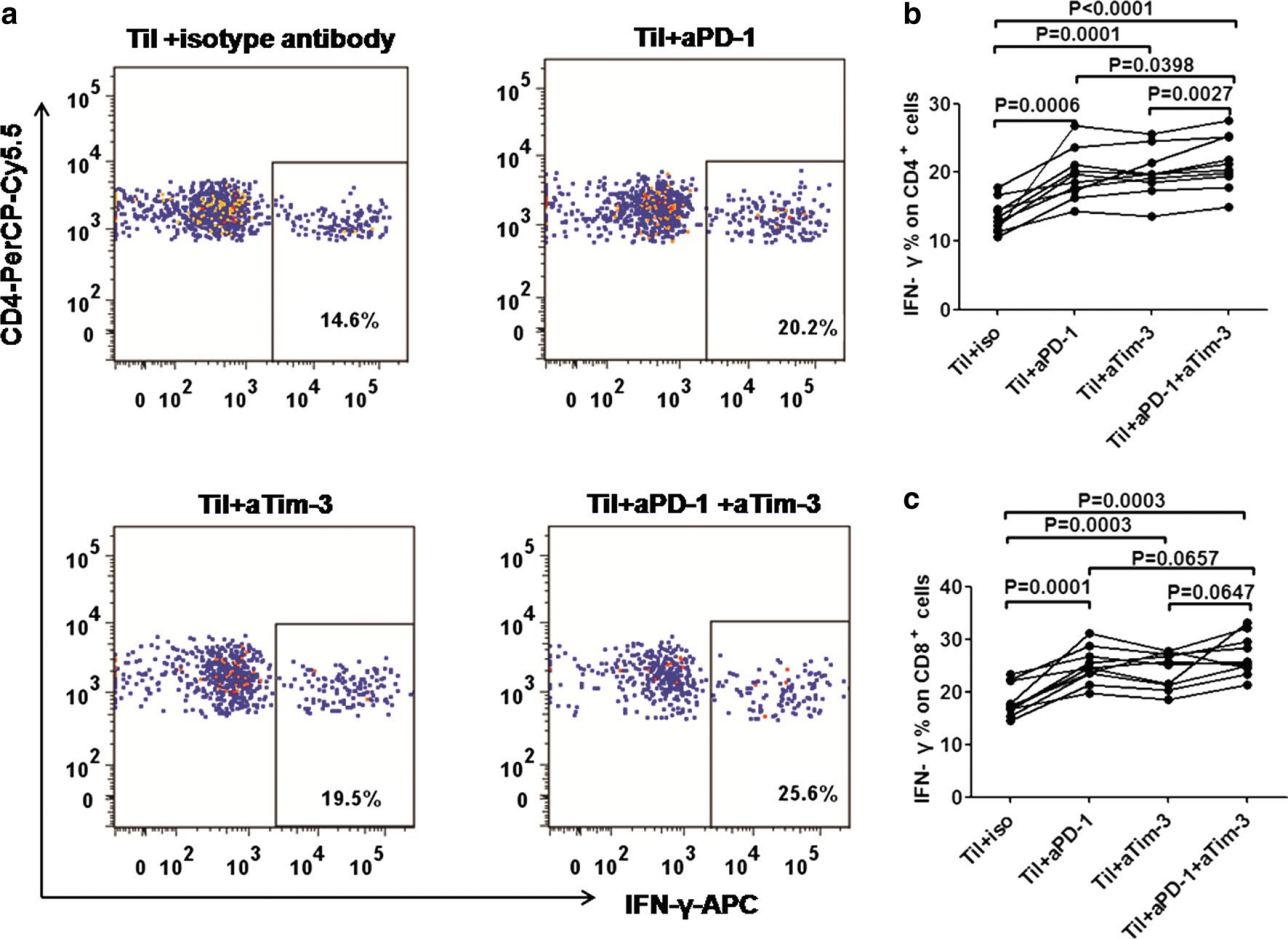
Effect of PD-1^+^ and/or Tim-3^+^ blockade on IFN-γ-producing CD4^+^ and CD8^+^ T-cells from tumor tissues. **a** Data were displayed as FMO-corrected values with subtraction of the individual co-stimulated control sample. IFN-γ staining showed TILs cytokine secretion following anti-CD3 stimulation and in the presence of blocking antibodies. **b** Dot plot graphs showing the increase of IFN-γ secretion of CD4^+^ T-cells from isotype control antibody to a PD-1^+^and/or a Tim-3^+^. **c** Dot plot graphs showing the increase of IFN-γ secretion of CD8^+^ T-cells from isotype control antibody to a PD-1^+^ and/or a Tim-3^+^. *p* values were calculated by using paired *t* test

IFN-γ is the main cytokine involved in the immune response. The capacity of IFN-γ induction in patients with GC (n = 10) following in vitro blockade of PD-1^+^ and/or Tim-3^+^ was examined. Anti-CD3-mAbs can stimulate T lymphocyte, and brefeldin A treatment facilitates the detection of IFN-γ. After stimulated with anti-CD3-mAbs and brefeldin A, inhibited PD-1^+^ significantly enhanced the production of IFN-γ by tumor-infiltrating CD4^+^/CD8^+^ T-cells in patients with GC as compared with FMO control (*p* = 0.0006, Fig. 4b for CD4^+^; *p* = 0.0001, Fig. 4c for CD8^+^). Tim-3 blockade also increased production of IFN-γ by TILs (*p* = 0.0001 for CD4^+^, *p* = 0.0003 for CD8^+^). Additionally, PD-1-PD-L1 blockade in combination with Tim-3-Tim-3-L blockade further enhanced the capacity of tumor-infiltrating CD4^+^ T-cells to produce IFN-γ as compared with anti-PD-1-mAbs group (*p* = 0.0398) or anti-Tim-3-mAbs group (*p* = 0.0027). On the other hand, combination of PD-1^+^ with Tim-3^+^ inhibition had no synergistic effects on IFN-γ induction of CD8^+^ T-cells. Then, the effect of PD-1^+^ and Tim-3^+^ inhibition on IFN-γ induction of nontumor-infiltrating CD4^+^ T-cells was examined. Interestingly, blockade of PD-1^+^ and/or Tim-3^+^ failed to improve nontumor-infiltrating CD4^+^ T**-**cells to produce IFN-γ, but combined PD-1^+^ with Tim-3^+^ inhibition had synergistic effects on IFN-γ induction of nontumor-infiltrating CD4^+^ T-cells (*p* = 0.0318, Additional file 3: Figure S3).

### Correlation of Tim-3^+^ PD-1^+^ CD4^+^/CD8^+^ T-cells with clinicopathological features

**Fig. 5:**
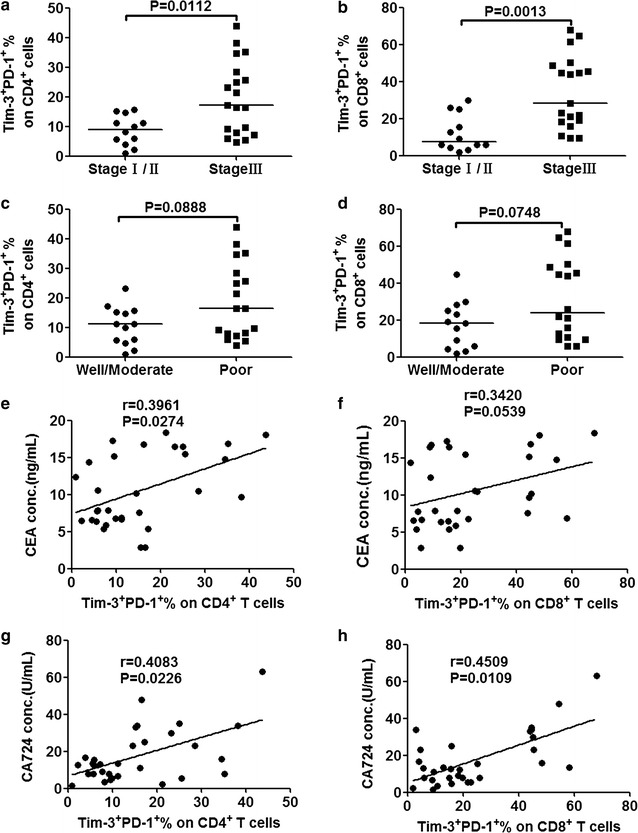
Elevated levels of tumor-infiltrating Tim-3^+^ PD-1^+^ CD4^+^/CD8^+^ T-cells in GC patients correlated with clinical stage. A significantly higher percentage of tumor-infiltrating Tim-3^+^ PD-1^+^ CD4^+^ (**a**) Tim-3^+^ PD-1^+^ CD8^+^ (**b**) T-cells were observed in cancer patients with stage III GC relative to stage I/II. Scatter plots of Tim-3^+^ PD-1^+^ among CD4^+^ (**c**) and CD8^+^ **(d**) T-cells percentage in well/moderate vs. poor differentiation patients. Percentage of tumor-infiltrating Tim-3^+^ PD-1^+^ CD4^+^/CD8^+^ T-cells from all patients were analyzed to correlate with the serum concentration (before treatment) of cancer biomarker CEA (**e** for CD4^+^, **f** for CD8^+^), and CA724 (**g** for CD4^+^, **h** for CD8^+^). Unparametric spearman correlation analysis was performed by GraphPad software. Bar denotes median in each group. *p* < 0.05 was considered as statistically significant

The association of tumor-infiltrating Tim-3^+^ PD-1^+^ CD4^+^/CD8^+^ T-cells with clinicopathological parameters was further analyzed in cancer patients. Patients were divided by clinical cancer stage. Significant differences were observed for the Tim-3^+^ PD-1^+^ CD4^+^/CD8^+^ percentage in stage III patients compared to stage I/II GC patients (17.2% vs. 9.02%, *p* = 0.0112, Fig. 5a for CD4^+^; 28.4% vs. 7.47%, *p* = 0.0013, Fig. 5b for CD8^+^). No correlation was present between the histological grade and Tim-3^+^ PD-1^+^ CD4^+^/CD8^+^ T-cells (Fig. 5c for CD4^+^; Fig. 5d for CD8^+^). Then, we analyzed the correlation between the Tim-3^+^ PD-1^+^ CD4^+^/CD8^+^ T-cells and serum concentration of cancer biomarker which was tested pre-treatment. The results showed no correlation between the percentage of Tim-3^+^ PD-1^+^ CD4^+^/CD8^+^ T-cells and the serum concentration of cancer biomarker CEA (Fig. 5e for CD4^+^; Fig. 5f for CD8^+^) or CA724 (Fig. 5g for CD4; Fig. 5h for CD8).

**Fig. 6:**
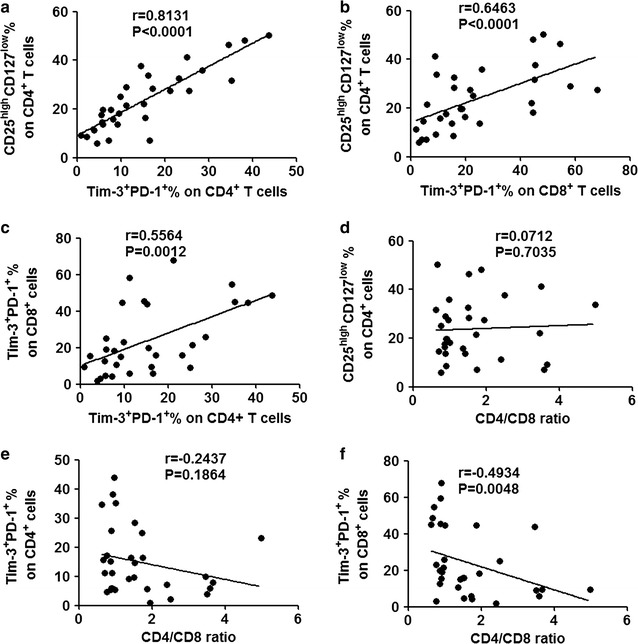
Elevated levels of tumor-infiltrating Tim-3^+^ PD-1^+^ CD4^+^/CD8^+^ T-cells in GC patients correlated with Treg. Percentage of tumor-infiltrating Tim-3^+^ PD-1^+^ CD4^+^/CD8^+^ T-cells from all patients were analyzed to correlate with Treg (**a** for CD4^+^, **b** for CD8^+^). The correlation of the percentage of tumor-infiltrating Tim-3^+^ PD-1^+^ CD4^+^ cells with Tim-3^+^ PD-1^+^ CD8^+^ (**c**). The correlation of the CD4^+^/CD8^+^ ratio with Treg (**d**), the percentage of tumor-infiltrating Tim-3^+^ PD-1^+^ CD4^+^ (**e**), and Tim-3^+^ PD-1^+^ CD8^+^ (**f**)

The correlation between Tregs and the tumor-infiltrating Tim-3^+^ PD-1^+^ CD4^+^/CD8^+^ T-cells was also examined. Significant correlations were observed for the Tim-3^+^ PD-1^+^ CD4^+^/CD8^+^ percentage and the frequency of Treg (R = 0.8131, *p* < 0.0001 for CD4^+^; R = 0.6463, *p* < 0.0001 for CD8^+^, Fig. 6), while no correlation was observed between the CD4^+^/CD8^+^ ratio and the percentage of Treg and the Tim-3^+^ PD-1^+^ CD4^+^/CD8^+^ percentage.

## Discussion

It has been reported that T lymphocytes played a critical role in controlling and eliminating cancer [3, 17, 18]. Researches revealed that tumor antigen would activate cytotoxic CD8^+^ T-cells which was enhanced by specific helper CD4^+^ T-cells [19, 20]. In our study, the GC tumors have higher percentages of CD4^+^ T-cells and a lower frequency of CD8^+^ T-cells compared with that in paraneoplastic tissues or blood, which indicated an increased CD4^+^/CD8^+^ ratio in tumors. In addition, the CD4^+^/CD8^+^ ratio in peripheral blood was significantly higher than that in matched paraneoplastic tissue. Our results showed that the T-cell subset distribution in patients with GC was different for varied tissues. The comparison is essential to the rational design for novel immuno-therapeutics strategy against GC. Previous reports showed that CD8^+^ T-cells prevailed over CD4^+^ T-cells in the tumor lesions derived from neuroblastoma patients, which meant that the CD4^+^/CD8^+^ ratio in tumors was lower than that in peripheral blood [20]. However, these results were quite different from ours. In addition, the percentage of CD4^+^ T-cells was decreased in livers when compared with the blood in inflammatory liver injuries and viral infections [21]. According to these studies, we conclude that different molecular mechanisms may cause the skewing of the CD4^+^/CD8^+^ T-cell ratio and the reason is not clear for the changes of CD4^+^ T-cells in GC patients, which needs further clinical investigations.

Previous studies have reported that there were increased frequency of effector and memory T-cells among virus-specific CD4^+^ T-cells during acute-resolving and chronic viral infections [18, 19]. In our research, the frequency of effector and memory T-cells among TILs and paraneoplastic T-cells elevated comparing with that of circulating T-cells, which was coincided with those in neuroblastoma patients. Fridman et al. found that the presence of CD8^+^ memory T-cells was associated with a favorable prognosis [22]. However, the relationship between the presence of CD4^+^ memory T-cells and prognosis in patients with GC has yet to be elucidated, and it will be explored in further researches.

In addition, regulatory T-cells can regulate antigens and suppress immunity to cancer [10]. However, the role of Tregs in anticancer immunity is more controversial. In some cancers such as ovarian carcinoma and HPV-associated patients, high numbers of tumor-infiltrating Tregs have shown poor outcomes [23, 24]. However, in colorectal cancer and lymphomas, Tregs have been reported as a positive prognostic factor [25, 26]. These findings implied that the role of Tregs might vary according to the type and etiology of the cancer. In GC, Choi et al. demonstrated that high level of Tregs among tumor-infiltrating CD4^+^ T-cells were favorable, but they only analyzed the Tregs in tumor tissue and peripheral blood from a healthy control [3]. On the contrary, Hennequin et al. showed that low infiltration of Tregs were associated with better relapse-free survival in patients with localized gastric cancer [11]. Moreover, there is still no definitive markers that used to define Treg. Instead, CD127^low^ expression, which is known to be highly enriched in regulatory CD4^+^ CD25^+^ T-cells, might be the most specific marker that is known and used so far [27–29]. Therefore CD4^+^ CD25^high^ CD127^low^ was used to assess Tregs in CD4^+^ T-cells of GC patients in our study. We discovered an elevated level of Tregs in peripheral blood from patients with GC when compared with those from HDs. Notably, the percentage of CD25^high^ CD127^low^ T regulatory cells among TILs were significantly higher than their counterparts in peripheral blood and paraneoplastic tissue, while the Tregs in peripheral blood has no significant difference as compared with that in Nils, which indicated that the GC milieu favors the accumulation of immunosuppressive Tregs at the tumor site.

Recent studies suggested that cells express only PD-1^+^ indeed retain Ag responsiveness in tumors, while only co-expression of PD-1^+^ and Tim-3^+^ identifies the most profoundly hypo-functional T-cells [14–16], and the blockade of them rejuvenates tumor-infiltrating CD8^+^ T-cells function in cancer patients. In present study, we compared the expression of PD-1^+^ and Tim-3^+^ on CD4^+^/CD8^+^ T-cells in blood circulation, tumor, and paraneoplastic tissues from patients with GC, and further tested the changes of IFN-γ production on T-cells after in vitro blockade of PD-1^+^ and/or Tim-3^+^ pathways. Our results showed that the frequencies of Tim-3^+^, PD-1^+^, and PD-1^+^ Tim-3^+^ cells among CD4^+^/CD8^+^ cells in circulation were significantly higher in GC patients than that in HDs. These data were consistent with previous reports of increased PD-1^+^ and Tim-3^+^ expression on T-cells in GC, which suggested that PD-1^+^ and Tim-3^+^ may be involved in immune evasion in GC patients [30–33]. In addition, PD-1^+^ and Tim-3^+^ expression has been described in gastric patients with *Helicobacter pylori* (*H. pylori*) infection, which is a major cause of gastric cancer [34, 35]. Studies showed that the removal of *H.* *pylori* infection could theoretically decrease the number of cases by 89% [36, 37]. Therefore, the interaction among *H. pylori* infection, the expression of PD-1^+^ and Tim-3^+^, and GC needs to be further explored.

It was also observed that the percentages of PD-1^+^, Tim-3^+^, and PD-1^+^ Tim-3^+^ cells among CD4^+^/CD8^+^ T-cells were significantly increased in the tumor tissues compared to their counterparts in matched peripheral blood and paraneoplastic tissues. Meanwhile, the percentages of Tim-3^+^, PD-1^+^, and PD-1^+^ Tim-3^+^ cells among CD4^+^ cells in paraneoplastic tissues were all significantly higher than those in peripheral blood. These results provided a solid foundation that TILs showed functional exhaustion in patients with GC, and supported the hypothesis that the tumor microenvironment played an important role in the up-regulation of inhibitory receptors [16, 38, 39]. Furthermore, our data indicated that the inhibition of PD-1^+^ and Tim-3^+^ significantly enhanced tumor-infiltrating CD4^+^/CD8^+^ T-cells IFN-γ secretion in patients with GC compared with the control group. These results were concordant with previous reports of impaired T-cells during viral infections and tumor growth and suggested that co-expression of Tim-3^+^ and PD-1^+^ was a marker of tumor-induced T-cell dysfunction [13, 38–40]. Previous researches have shown that the combination of Tim-3^+^ blockade with PD-1^+^ pathway blockade was remarkably more effective in colon carcinoma, acute myelogenous leukemia, and melanoma models than with blockade of either the Tim-3^+^ or PD-1^+^ pathway alone [41, 42]. In this study, we also observed that combined PD-1^+^ and Tim-3^+^ inhibition had a synergistic effect on CD4^+^ T-cells’ IFN-γ secretion, which was in an agreement with Smyth and Cunningham’s study [43]. But combined PD-1^+^ and Tim-3^+^ inhibition did not have synergistic effects on IFN-γ induction of CD8^+^ T-cells. This may be caused by the frequency of Tim-3^+^ PD-1^+^ T-cells occupying almost 90% of Tim-3^+^ CD8^+^ T-cells. In addition, although blockade of PD-1^+^ or Tim-3^+^ failed to improve the ability of nontumor-infiltrating CD4^+^ T**-**cells to produce IFN-γ, the combined PD-1^+^ and Tim-3^+^ inhibition had synergistic effects on IFN-γ induction of nontumor-infiltrating CD4^+^ T-cells. These results could be explained by following points:

1. The hierarchical co-regulation of multiple negative regulatory pathways on CD4^+^ and CD8^+^ T-cells [44];
2. The complex interactions between the inhibitory pathways during long-term in vitro conditions; and
3. The blocking Tim-3/galectin-9 interactions complementary to PD-1^+^ pathway inhibition [45].

## Conclusion

According to abovementioned results, combination therapies of the immune checkpoint inhibitors with other targeted agents were alternative for GC patients.

One novel finding was that the percentages of Tim-3^+^ PD-1^+^ CD4^+^/CD8^+^ among TILs were associated with Tregs and clinical stages, which suggested that Tregs may induce the expression of PD-1^+^ and Tim-3^+^. A significant difference was observed between patients with advanced tumors and patients with stage I/II, which indicated that the exhausted T-cells may stimulate tumor progression.

In conclusion, to the best of our knowledge, this is the first study to characterize the subset composition and functional properties of multiple inhibitory molecules on TILs and the correlation of Tim-3^+^ PD-1^+^ CD4^+^/CD8^+^ T-cells in tumors with clinic opathological features and Treg in GC patients. Our findings provide new insights into the mechanisms underlying T-cell dysfunction and allow for future immunotherapeutic strategies in GC.

## Additional files

**Additional file 1: Figure S1.** Frequencies of TEM and TCM on CD4^+^ and CD8^+^ T-cell. (A) Flow cytometry results; (B) frequencies of TEM and TCM on CD4^+^; Frequencies of TEM and TCM on CD8^+^.

**Additional file 2: Figure S2.** Expression of inhibitory molecules Tim-3^+^ and PD-1^+^ on CD8^+^. (A) Flow cytometry results; (B) frequency of Tim-3^+^ cells among CD4^+^ and CD8^+^ cells in circulation of GC patients and HDs; (C) PD-1^+^ cells on T-cells in circulation of GC patients and HDs; (D) median percentage of PD-1^+^ Tim-3^+^ cells among CD4^+^ and CD8^+^ in GC patients and HDs; E percentage of Tim-3^+^ cells in different tissues; F Expression level of PD-1^+^ for CD8^+^; G PD-1^+^ Tim-3^+^ cells among T-cells.

**Additional file 3: Figure S3.** Effects of PD-1^+^ and Tim-3^+^ inhibition on IFN-γ induction. (A) Flow cytometry results; (B) IFN-γ induction on CD4^+^ cells.
