# Supplementary figures and images for: Tumor-infiltrating CD4+ T cells in patients with gastric cancer
Source: Cancer Cell Int. 2017 Dec 2;17:114. doi: 10.1186/s12935-017-0489-4 (PMC5712164; doi:10.1186/s12935-017-0489-4)

## Slide 1
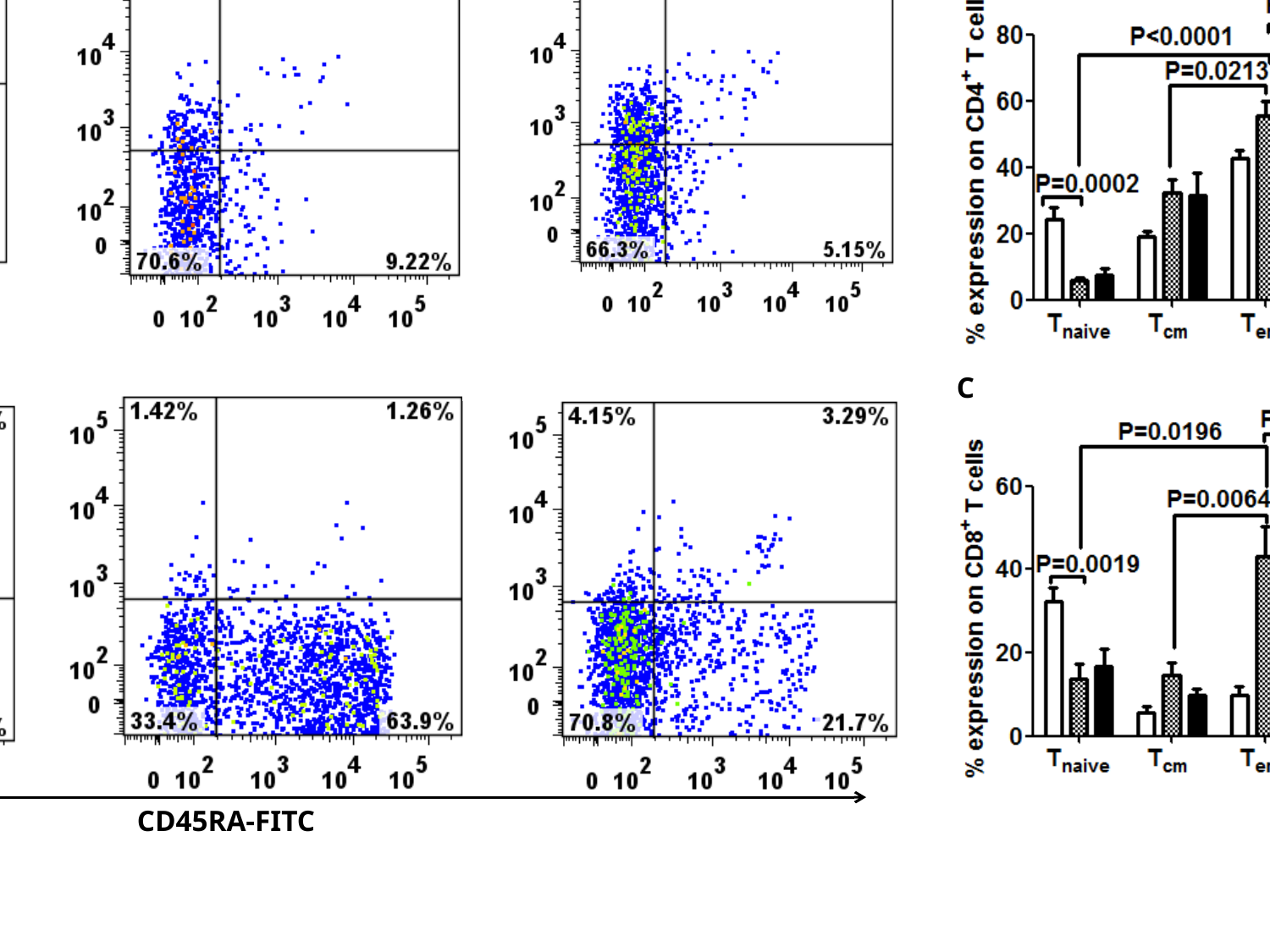

Fig S1
A Blood Tumor Nontumor B
 CD8 CCR7-PE-Cy7 CD4
 CD45RA-FITC
C

Supplement: Supplementary file 1 — Additional file 1: Figure S1. Frequencies of TEM and TCM on CD4+ and CD8+ T-cell. (A) Flow cytometry results; (B) frequencies of TEM and TCM on CD4+; Frequencies of TEM and TCM on CD8+. [file 12935_2017_489_MOESM1_ESM.pptx]

## Slide 1
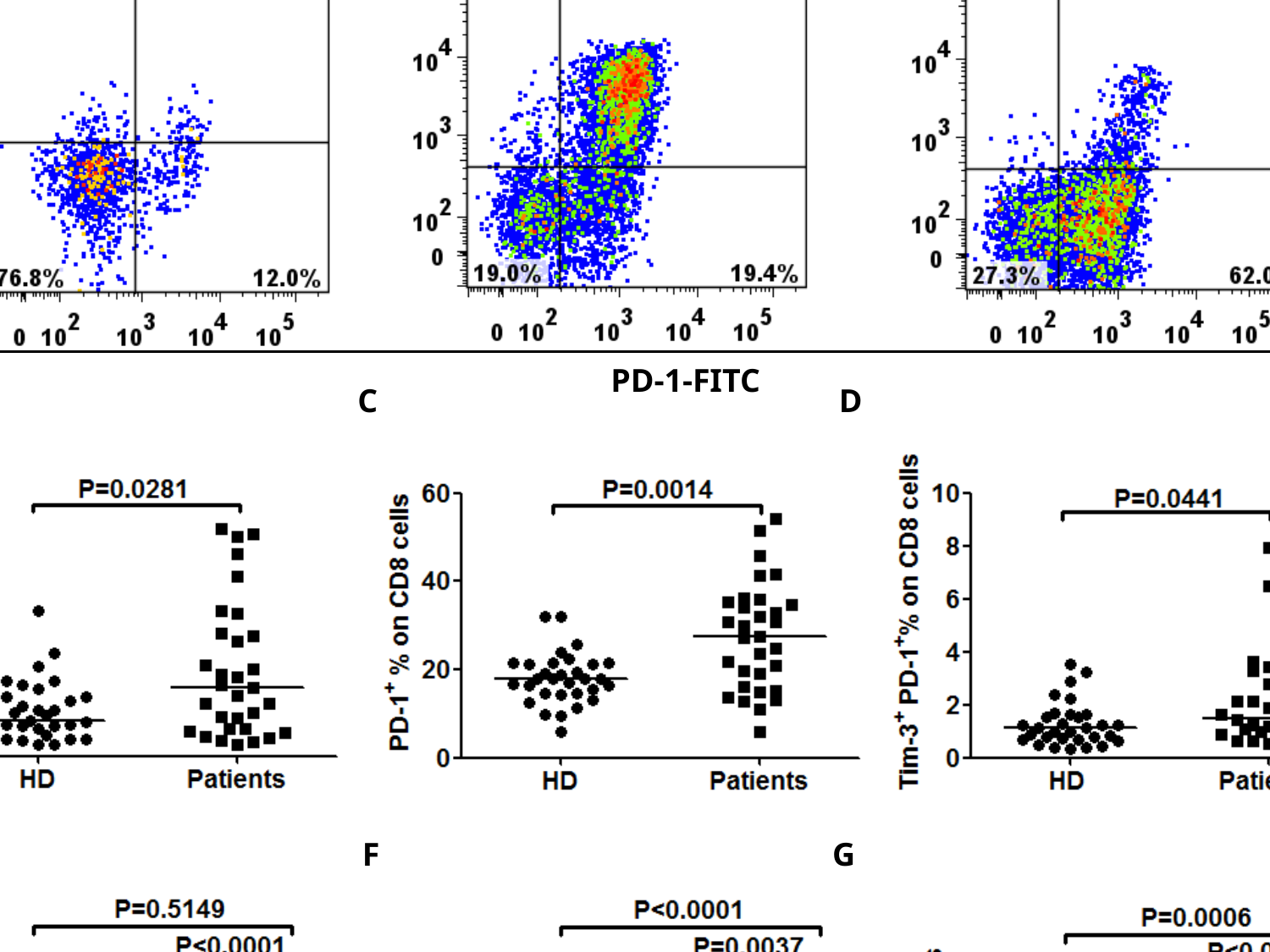

Fig S2
A Blood Tumor Nontumor
Tim-3-PE
PD-1-FITC
B C D
E F G

Supplement: Supplementary file 2 — Additional file 2: Figure S2. Expression of inhibitory molecules Tim-3+ and PD-1+ on CD8+. (A) Flow cytometry results; (B) frequency of Tim-3+ cells among CD4+ and CD8+ cells in circulation of GC patients and HDs; (C) PD-1+ cells on T-cells in circulation of GC patients and HDs; (D) median percentage of PD-1+ Tim-3+ cells among CD4+ and CD8+ in GC patients and HDs; E percentage of Tim-3+ cells in different tissues; F Expression level of PD-1+ for CD8+; G PD-1+ Tim-3+ cells among T-cells. [file 12935_2017_489_MOESM2_ESM.pptx]

## Slide 1
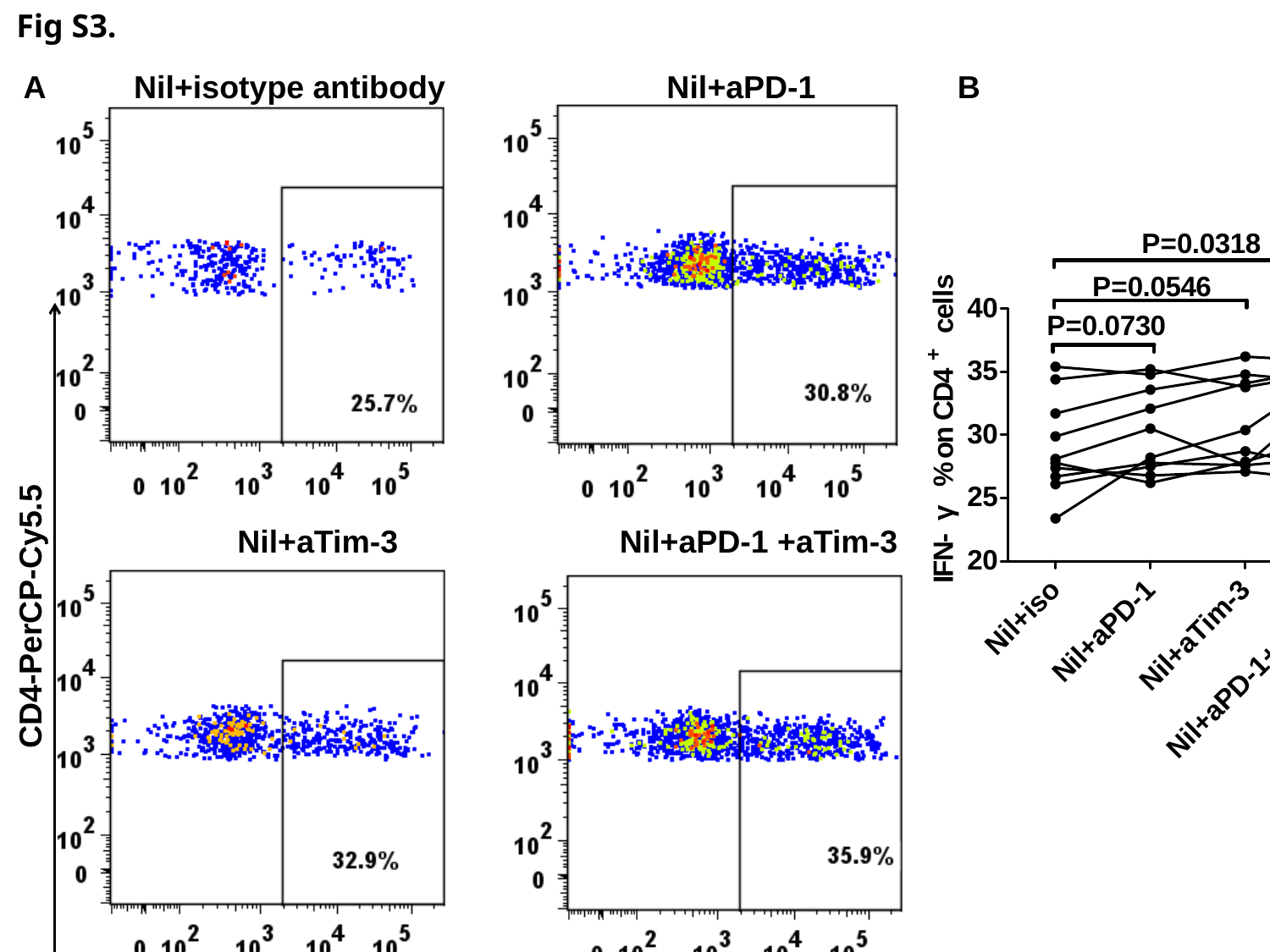

Fig S3.
A Nil+isotype antibody Nil+aPD-1 B
 Nil+aTim-3 Nil+aPD-1 +aTim-3
CD4-PerCP-Cy5.5
IFN-γ-APC

Supplement: Supplementary file 3 — Additional file 3: Figure S3. Effects of PD-1+ and Tim-3+ inhibition on IFN-γ induction. (A) Flow cytometry results; (B) IFN-γ induction on CD4+ cells. [file 12935_2017_489_MOESM3_ESM.pptx]
